# Continued Malarial Fever

**Published:** 1901-11

**Authors:** J. R. G. Howell

**Affiliations:** Dothan, Ala.


### CONTINUED MALARIAL FEVER.*

By J. R. G. HOWELL, M.D.,

DOTHAN, ALA.

I understand that when the term continued malarial fever is used that our attention is called to that type of malarial fever which has been called by some typho-malarial fever, and by others slow fever. Some writers of to-day object to the former term on the ground that it is misleading; they say that the fever is not a mixed fever at all, but that it is either typhoid fever with typhoid bacillus in the stools, or it is malarial fever with the plasmodia malariæ showing in the red blood-corpuscles under the microscope. Others claim the fever is malarial in the beginning and typoid in the ending. Not being a skilled microscopist I am unable to say who is right or who is wrong. Some object to the term slow fever; they say that the fever is slow only on account of a slow doctor. It is said that if a fast doctor had the case in charge that the fever would be of short duration, and there would be no such thing as long-continued cases of malarial fever. I do not like this much, for I am a slow one myself in these cases. From my experience I am thoroughly convinced that prudence is as essential in the beginning of these fevers as anywhere in the practice of medicine. Heroic treatment may contribute to a fatal termination. Let us notice for a moment the term malaria. It means bad air. It seems by this definition that we must contract the poisoning through the air if we get malarial fever. I look on this as misleading. I regard continued malarial fever as an enteric fever and most frequently contracted through what we eat and drink. I will admit, however, that miasmatic influences may produce continued malarial fever with all of its intestinal lesions. I further regard it as quite different from typhoid fever, which is strictly an enteric fever, and more widely different from remittent fever, which Dr. Flint thinks it so similar to. It is my impression that most authors now agree that all remittent fevers and all intermittent fevers are malarial.

---

*Read before the Henry County Medical Society October 1, 1901.

This may or may not be the case, as we use the term in a broad sense and not in a restricted sense. I now intend to leave all these differences with you and the microscopist, and go directly into the description of, and treatment of, what I believe to be continued malarial fever. Most cases come on with a chill; not a chill as is common in intermittent type ot malarial fever, but one in which we have cold extremities and general stupid feeling with a gradual rise of temperature, the temperature rising gradually to 102, and we do not have the stage of excessive perspiration that is usually present in the intermittent type of fever; neither do we have the rapid rise of temperature to 105 as is common in such cases. This chill is not present in all cases, neither is the chill the initial symptom in all cases, for it occasionally comes on the third or fourth day of the fever, but most likely the first day you see the patient, or, perhaps, what is worse, the day before you see the patient. This chill may return each day for quite a while, and in a few cases I have seen it return up to the twenty-first day of the fever, and later assuming the form of a rigor.

The temperature assumes rather a remittent type, rising higher at certain hours in the day, and then remitting at certain other hours, but gradually rising higher each day until it reaches 103, 104, or 105, as the case may be. It will reach its highest pitch much earlier and remain there very much longer than typhoid fever. It is a further noticeable fact that when it turns down it makes the descent in a much shorter time than the typical typhoid. The pulse follows the temperature up and reaches about 120 per minute in very many cases, and comes down to normal in advance of the temperature at the end of the fever, if it ends in recovery. The pulse, however, will not come down with the temperature when the disease is not nearing the end. I have therefore learned to watch the pulse with great anxiety, as thereby I am enabled to see much further into the future of my case. In a few rare cases we have a sudden onset of the disease with the temperature rising to 105 in the first twenty-four hours. I now remember a patient who spent the day up feeling quite well, and at 12 o'clock at night I was called to find her temperature 105 and circulation 125. This case lasted seven weeks. The tongue presents rather a typical appearance. The edges are red and the border has a rather light-colored coat; while the center of the tongue is covered with a brown coating. All around on the white border there are strawberry spots very much like those common in catarrhal fever. In the beginning the tongue is not very dry, but as the disease advances the coating disappears; the tongue becomes quite red and dry, and if certain lines of treatment are adopted the tongue will become exceedingly dry, and cracks will appear in it; while the teeth will be covered with sordes. There is anorexia from the beginning of the fever. Constipation is frequent, and nausea is a common symptom. The urine is scanty, high colored, and the specific gravity often reaches 1030. There is very seldom albumin in the urine in the early stages of the disease, but as the fever declines the albumin will appear and be constant in very many cases for some time after convalescence sets in; in fact I have found it after the patient had been up for four weeks. There is some headache and some aching of the back and limbs, but not that intense form that we have in other types of malarial fever. There is not that photophobia that is present in typhoid fever; neither do we find the extreme indifference that is so marked with the typhoid fever patients. On a physical examination we often find an abnormally large liver, or a hypertrophied spleen. After the seventh day we begin to find enlargement and tympanites of the abdomen. We do not find that localized tenderness and tympanites confined to Peyer's glands, but we find a gurgling and tympanitic condition throughout the entire abdominal cavity. The rose spots so common in typhoid fever are absent. Now for the treatment. Here is where I have been told that my shortcomings are more prominent than elsewhere. I have been told for the past ten years, both by personal friends and those writing in journals, that if I would use calomel and quinine with sufficient heroism, that my cases of so-called continued malarial fever would terminate in about one week, or less time. I wish to state just here that I used it as prescribed by those physicians who claim to be able to abort the fever, and the disease went on for three or four weeks seemingly at its own sweet will. The only difference that I could see in this plan of treatment and others that I have adopted is that where large quantities of quinine were given there was greater disturbance of the nervous system and stomach. I found numbers of cases of fevers that I did break up very quickly, but they were remittent fevers, intermittent fevers, and ephemeral fevers. I have grown to be almost as doubtful of some of my brethren in their diagnosis as they are of me in my treatment; in fact it has almost reached the point where I look upon them with the same suspicion as that entertained for those who cure gonorrhea in three days. I think in the treatment of continued malarial fever, like the treatment of typhoid fever, it is very much easier to give too many remedies, and do too much, than it is to do too little. There are quite a number of remedies that I once used that I have now discarded altogether. I once used turpentine emulsion quite extensively, also the chlorine mixture, but I do not now use either preparation on account of the dry mouth, dry tongue, and sordes on the teeth produced by these remedies. I do not think it a good idea to give alcohol in any of its forms, nux vomica, or arsenic except in very rare cases.

You will be tempted to call me a semi-hydropath in my treatment of this disease, for I allow my patients to drink considerable water, have them sponged off frequently, and cause large quantities of water to be injected into the colon. There is no line of treatment of this disease that will meet the emergencies in all cases, for, in this disease as in all others, we must meet the indications as they come up. There is, however, a treatment that I use in most cases, and I will outline it just here. For the first few days while the constipation lasts, and while the brown coating remains on the tongue I give calomel and soda in small doses. For the first day I give two grains of calomel and soda each every two hours until three doses are given. Then on the third day if not contraindicated I give one grain of calomel and soda each every two hours until three doses are given. Then on the fifth day if I see good reason for doing so I give one-half grain of calomel and soda each every two hours until three doses are given. If needed I follow each of these courses with sul. magnesia. I seldom use any more purgatives, for by this time the tongue is cleaned off and the bowels have become rather active, and enemas are used from this time on. I begin giving quinine the first day I see the patient, and give six grains of the bisulphate every three hours until twenty-four grains are given each day. This is continued for about four or five days, after which the quantity of quinine is reduced very materially, or discontinued altogether, as the case may demand.

When I have discontinued the quinine, and sometimes before I leave it off, I begin with a four-ounce mixture composed of one-half ounce of nitro-muriatic acid dilute, and three and one-half ounces of elixir of lactopeptine, and give one teaspoonful every four hours while the patient is awake. This sometimes causes the bowels to be too active, and in such cases I leave it off entirely. I control the fever with phenacetin and cold water enemas. Sponging is sometimes resorted to, but that is mostly to excite activity in the skin and not with the hope of an immediate reduction of the fever. I do not give large doses of phenacetin, nor allow a frequent repetition of the dose. In the beginning of the fever two six-grain doses during twenty-four hours is sufficient; after the tenth or twelfth day I seldom give over three grains at a dose, and generally give only one dose a day. There are but two articles of nourishment that have served me well in these troubles; they are buttermilk and beef tea from Mosquera's beef jelly; the peptonoids, milk punch, soups of various kinds and egg-nogs, have all impressed me as being inferior to the first two articles mentioned. I think that the milk punch and egg-nogs not only fail to be useful, but are actually harmful in a vast majority of cases. I employ all the hygienic measures possible, not only for the good of others of the family, but for the patient himself. Autoinfection may not be possible in these cases, but pure air is certainly the best for the patient. Hoping that what I have said may provoke a general discussion that will prove beneficial to us all, I will close this paper.

